# Prostaglandins, Masculinization and Its Disorders: Effects of Fetal Exposure of the Rat to the Cyclooxygenase Inhibitor- Indomethacin

**DOI:** 10.1371/journal.pone.0062556

**Published:** 2013-05-03

**Authors:** Afshan Dean, William Mungall, Chris McKinnell, Richard M. Sharpe

**Affiliations:** MRC Centre for Reproductive Health, The Queen's Medical Research Institute, University of Edinburgh, Edinburgh, Scotland, United Kingdom; Clermont Université, France

## Abstract

Recent studies have established that masculinization of the male reproductive tract is programmed by androgens in a critical fetal ‘masculinization programming window’ (MPW). What is peculiar to androgen action during this period is, however, unknown. Studies from 20 years ago in mice implicated prostaglandin (PG)-mediation of androgen-induced masculinization, but this has never been followed up. We therefore investigated if PGs might mediate androgen effects in the MPW by exposing pregnant rats to indomethacin (which blocks PG production by inhibiting cyclooxygenase activity) during this period and then examining if androgen production or action (masculinization) was affected.

Pregnant rats were treated with indomethacin (0.8 mg/kg/day; e15.5–e18.5) to encompass the MPW. Indomethacin exposure decreased fetal bodyweight (e21.5), testis weight (e21.5) and testicular PGE_2_ (e17.5, e21.5), but had no effect on intratesticular testosterone (ITT; e17.5) or anogenital index (AGI; e21.5). Postnatally, AGI, testis weight and blood testosterone were unaffected by indomethacin exposure and no cryptorchidism or hypospadias occurred. Penis length was normal in indomethacin-exposed animals at Pnd25 but was reduced by 26% (p<0.001) in adulthood, an effect that is unexplained.

Our results demonstrate that indomethacin can effectively decrease intra-testicular PGE_2_ level. However, the resulting male phenotype does not support a role for PGs in mediating androgen-induced masculinization during the MPW in rats. The contrast with previous mouse studies is unexplained but may reflect a species difference.

## Introduction

Masculinization by androgens is a pivotal event in the development of a phenotypic male, and mild disorders of this process (hypospadias, cryptorchidism) are common in human males at birth [1]–[3]. In rats, there is a critical period, the masculinization programming window (MPW), during which sufficient testosterone, produced by the fetal testis, ‘programs’ the male reproductive tract so that it can differentiate and grow normally after the MPW [4]. Any impairment of androgen action during the MPW results in smaller reproductive organs and/or abnormal function as well as decreased anogenital distance (AGD) [4],[5]. AGD, the distance between the anus and genitalia, is about twice as long in male rats as in females and provides a life-long readout of androgen action during the MPW [4]. A similar MPW may exist in humans, within the period 8–14 weeks of gestation [4], and AGD is approximately twice as long in males as in females at birth [6],[7] and is therefore suggested to provide a similar read-out of fetal androgen exposure in the MPW as in the rat [6]–[9].

The molecular mechanisms and androgen pathways triggered during the MPW remain to be elucidated. Prostaglandins (PGs) are potentially implicated based on early studies by Gupta [10],[11] in which treatment of pregnant mice with cyclooxygenase (COX) inhibitors (aspirin, indomethacin) during the period of the MPW was shown to reduce male AGD, with the effect clearly downstream of androgen action. PGs are lipid compounds derived from arachidonic acid [12]. They mediate many important biological functions, acting in a paracrine or autocrine fashion. COX1 and COX2 are fundamental enzymes in the synthesis of PGs from arachidonic acid. PGs are recognized to be involved in various reproductive functions such as ovulation and implantation [13],[14] and induction of labor [15],[16].

There is further evidence supporting a role for PGs in mediating aspects of androgen action in the MPW. Four epidemiological studies have shown that use of paracetamol (acetaminophen), with/without other painkillers that interfere with PG production (such as aspirin), by women in pregnancy during the period of the proposed MPW, significantly increase the risk of cryptorchidism in sons [17]–[20]. Rat studies have shown that inhibition of androgen action in the MPW increases incidence of cryptorchidism [4],[21] and, consistent with this, paracetamol and/or other prostaglandin inhibitors (aspirin, indomethacin) have been shown to reduce testosterone production in vitro by fetal rat testes [22]. Viewed together, the aforementioned data makes a strong case for the possibility that prostaglandins mediate aspects of androgen action in the MPW, and with human health relevance. The objective of the present study was to directly test this possibility by investigating in the rat if inhibition of PG production by indomethacin during the MPW was able to interfere with normal masculinization and thus to induce male reproductive disorders such as hypospadias or cryptorchidism. These disorders are associated with a decrease in androgen action during the MPW resulting in a decreased AGD [4].

## Methods

### Animals and Treatments

For all studies, animals were treated humanely and with regard for alleviation of suffering. Studies were performed according to the Animal (Scientific Procedures) Act 1986 following specific approval by the UK Home Office. Studies were conducted under Project Licence PPL 60/3914 following review by the University of Edinburgh Animal Research Ethics Committee. Wistar rats were maintained according to UK home office guidelines and had free access to fresh tap water and soy free diet (SDS; Dundee, Scotland). Time-matings were established by the presence of a vaginal plug and this was defined as embryonic day (e) 0.5. To investigate if exposure to indomethacin can affect fetal masculinization, pregnant animals were exposed to indomethacin (Sigma-Aldrich, UK) during the MPW (e15.5–e18.5) to determine if PGs are involved in androgen programming during this time. Animals that were killed on e17.5 received treatment up until e16.5 whereas those sampled on e21.5 received treatment until e18.5. The indomethacin dose of 0.8 mg/kg was selected following preliminary studies that investigated a number of doses (2, 1 or 0.8 mg/kg) but which determined that the two higher doses resulted in unacceptable litter loss and/or severe maternal intra-gastric bleeding. Control dams were injected with corn oil daily in the same time window (e15.5–e18.5). Data for male offspring collected in fetal life were from a minimum of 6 litters per treatment group and for postnatal studies from a minimum of 3 litters.

### Tissue recovery and measurements

Control and treated rat dams were killed on either e17.5 (during the MPW), e21.5 or allowed to give birth, and resulting offspring then killed on either postnatal day (Pnd) 25 ( =  early puberty) or 75 ( =  adults). For fetal studies, pregnant dams were killed by inhalation of CO_2_ followed by cervical dislocation, fetuses were removed, and placed in ice-cold PBS (Sigma-Aldrich). E21.5 fetuses were weighed and AGD measured using digital calipers (Faithfull Tools, Kent, UK) and the fetuses then decapitated. The gonads were removed by microdissection from both e17.5 and e21.5 fetuses. Tissue was either stored at−80°C for PGE_2_ or intratesticular testosterone ELISA assays or fixed in Bouin's fixative for 1 h, then transferred to 70% (v/v) ethanol and processed into paraffin wax blocks using standard procedures and an automatic tissue processor.

Postnatal animals were killed by inhalation of CO_2_ followed by cervical dislocation. Blood was collected from the heart into a heparinized syringe and the plasma separated and stored at−20°C. Bodyweight and AGD were measured before dissection of the penis and gonads, which were weighed. The length of the dissected penis was measured by digital callipers as described previously [23]. Tissue was fixed in Bouin's fixative for 6 h before being transferred to 70% (v/v) ethanol and embedded in paraffin wax as above.

### Prostaglandin E_2_ (PGE_2_) measurement

PGE_2_ was measured in the fetal gonads from control and indomethacin-treated groups to determine if effective exposure of the fetus had occurred. For collection of e17.5 samples treatment protocol described above was followed, whereby dams were treated on e15.5, e16.5 and e17.5 but then pups were removed 4 h after maternal injection on e17.5, gonads dissected and frozen at−80°C. Although this showed significant suppression by indomethacin of testicular PGE_2_ levels on e17.5 (ie during the MPW), to provide further reassurance that effective exposure of the fetus was occurring we undertook a similar study outside of the MPW on e21.5. For this study, dams were treated with indomethacin only on e20.5 and e21.5 and culled 4 h after the final treatment (see Fig. S1). Gonads from each animal were pooled and homogenized in 0.05 mM Tris/HCL PH 7.4 and PGE_2_ levels were determined in each sample using Detect X Prostaglandin E_2_ Enzyme Immunoassay kit, according to manufacturer's instructions (Arbor Assays, Michigan, US). PGE_2_ levels were read using an optical microplate reader (Labsystems, MutiSKan Ex, UK) and results analyzed using MasterPlex™ ReaderFit software (MiraiBio Group, Hitachi Ltd).

### Testosterone measurements

Fetal intratesticular testosterone levels were measured by competitive radioimmunoassay (RIA). All other details were as described previously [24]. Pnd25 and Pnd75 plasma levels of testosterone were measured using an enzyme-linked immunosorbent assay adapted from an earlier RIA method. This has been described in detail previously [25].

### Statistics

Data are expressed as means ±SEM. All analyses used GraphPad Prism. Rat data was analyzed using one-way ANOVA followed by the Bonferroni post-test or using students t-test as appropriate (version 5; GraphPad Software Inc., San Diego, CA, USA). The presented rat data used each animal as the unit rather than the litter, but re-analysis of the data using litter means did not alter any of the main findings. As there were significant treatment effects on bodyweight that may affect AGD, the latter was divided by the cube root of bodyweight to determine the anogenital index (AGI), and this was used in graphs and for statistical analyses. Data for testosterone was log-transformed prior to analysis to remove heterogeneity of variance.

## Results

### Fetal effects of exposure to indomethacin

**Figure 1:**
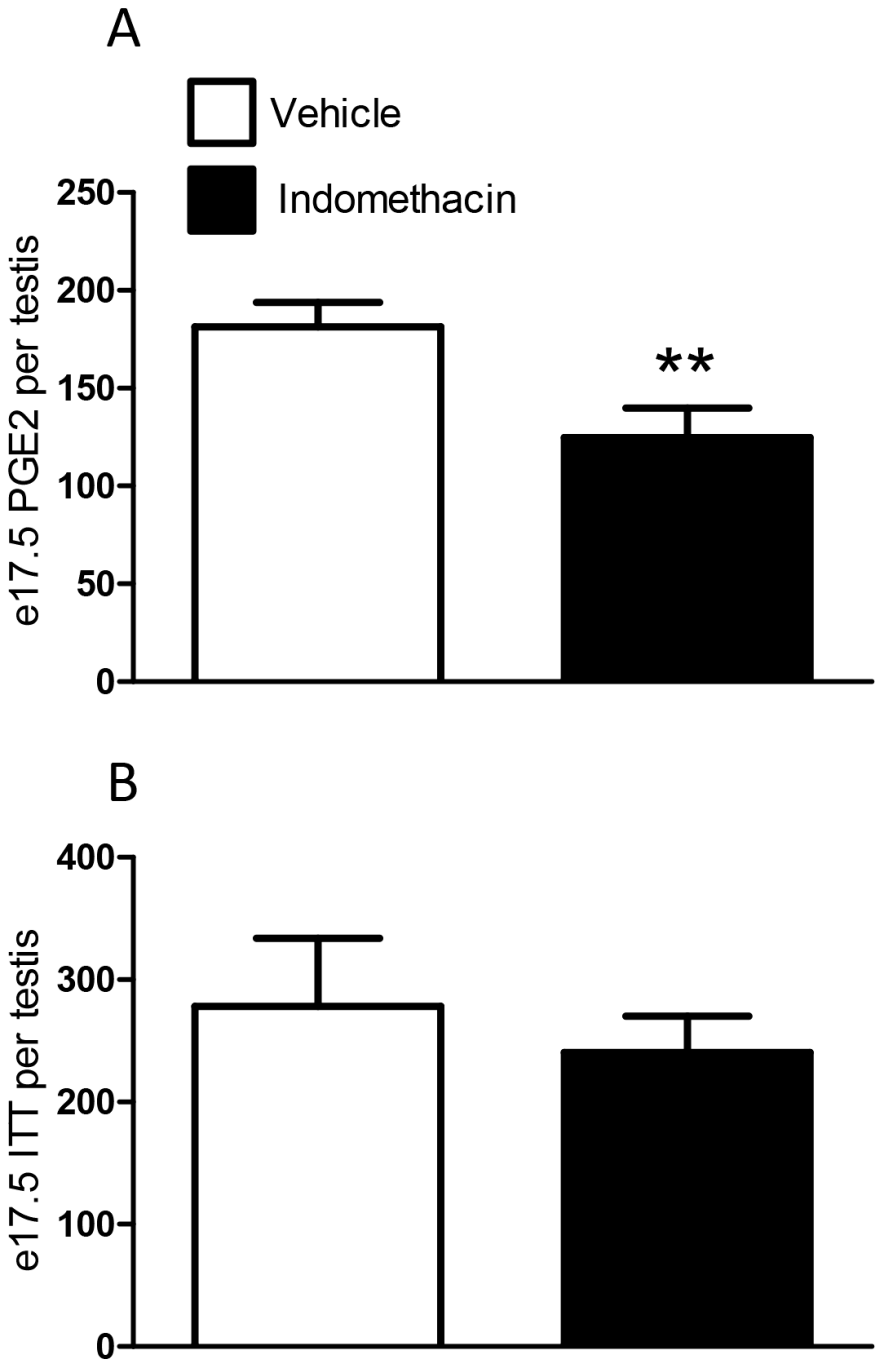
Effects of maternal exposure to vehicle or indomethacin (0.8 mg/kg/day) on (A) testicular PGE_2_ levels at e17.5 and (B) intra-testicular testosterone content at e17.5. Values are means ± SEM for N = 5 for the top graph and N = 7–28 for the bottom graph, from a minimum of 3 litters. **p<0.01, in comparison with respective control.

**Figure 2:**
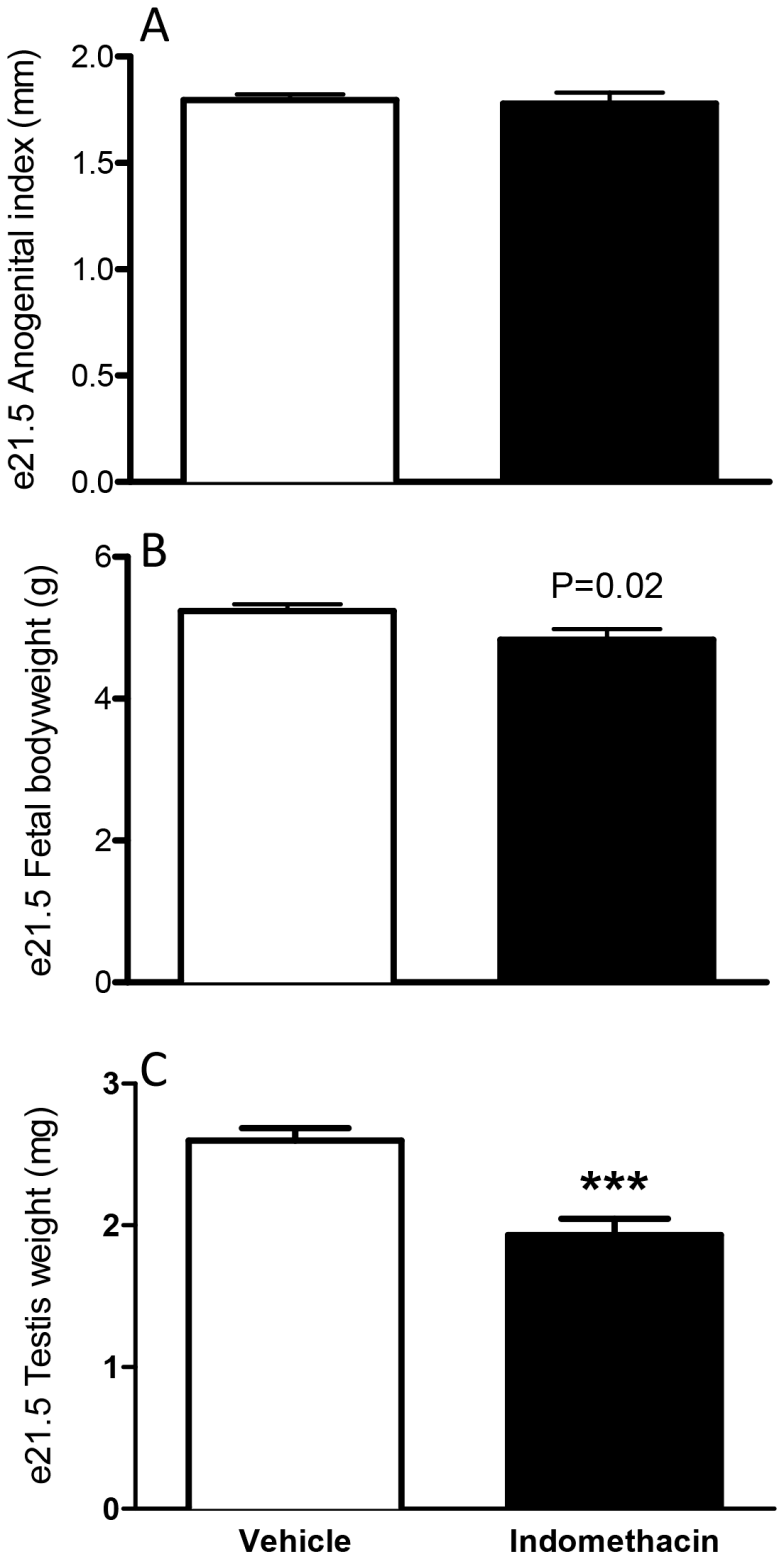
Effects of maternal exposure to vehicle or indomethacin (0.8 mg/kg/day; e15.5–e18.5) on anogenital index (A), bodyweight (B) and testis weight (C) at e21.5. Values are means ± SEM for N = 7–45 from a minimum of three litters ***p<0.001 in comparison with respective control.

At 4 h after maternal indomethacin treatment, PGE_2_ levels in the fetal testis were significantly decreased at e17.5 (during the MPW) when compared to controls (Fig. 1A), indicating effective exposure of the fetus. To provide further reassurance on this, we established separately that our indomethacin treatment protocol suppressed PGE_2_ levels in the fetal testis on e21.5 (Fig S1). However, fetal exposure to indomethacin during the MPW did not significantly alter intratesticular testosterone (ITT) levels at e17.5, in comparison to vehicle exposed animals (Fig 1B). Testosterone levels/androgen action during the MPW is reflected in AGD/AGI measurements from e21.5 onwards [4],[5]. In keeping with the data for e17.5 ITT, fetal indomethacin exposure during the MPW did not affect AGI at e21.5 (Fig. 2A), but did decrease fetal body weight of e21.5 male fetuses (Fig. 2B). Furthermore, exposure to indomethacin caused a significant decrease (27%) in fetal testis weight at e21.5 and this decrease occurred despite the fact that indomethacin treatment had ceased after e18.5 (Fig. 2C). If indomethacin treatment (0.8 mg.kg/day) was administered for a longer period (e15.5–e20.5), a similar decrease was found in testis weight (data not shown).

### Postnatal effects of fetal exposure to indomethacin

Offspring were evaluated on Pnd25 and Pnd75. In utero exposure to indomethacin did not result in hypospadias or cryptorchidism in any offspring (for vehicle N = 0/56; for indomethacin 0/26).

**Figure 3:**
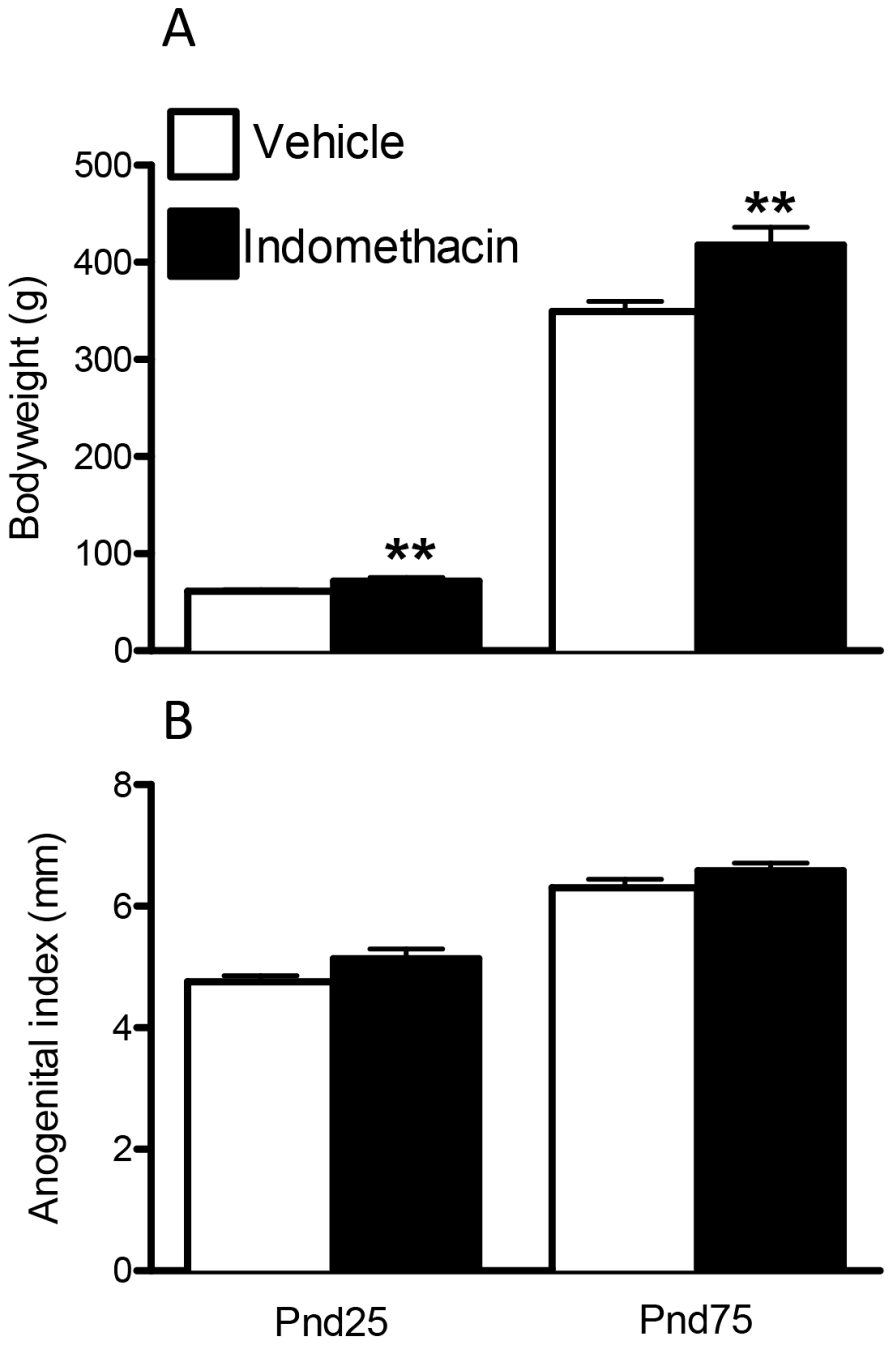
Effects of maternal exposure to vehicle or indomethacin (0.8 mg/kg/day; e15.5–e18.5) on bodyweight (A), and anogenital index (B) at Pnd25 and Pnd75 (adulthood). Values are means ± SEM for N = 6–20 from a minimum of three litters. **p<0.01 in comparison with respective control.

**Figure 4:**
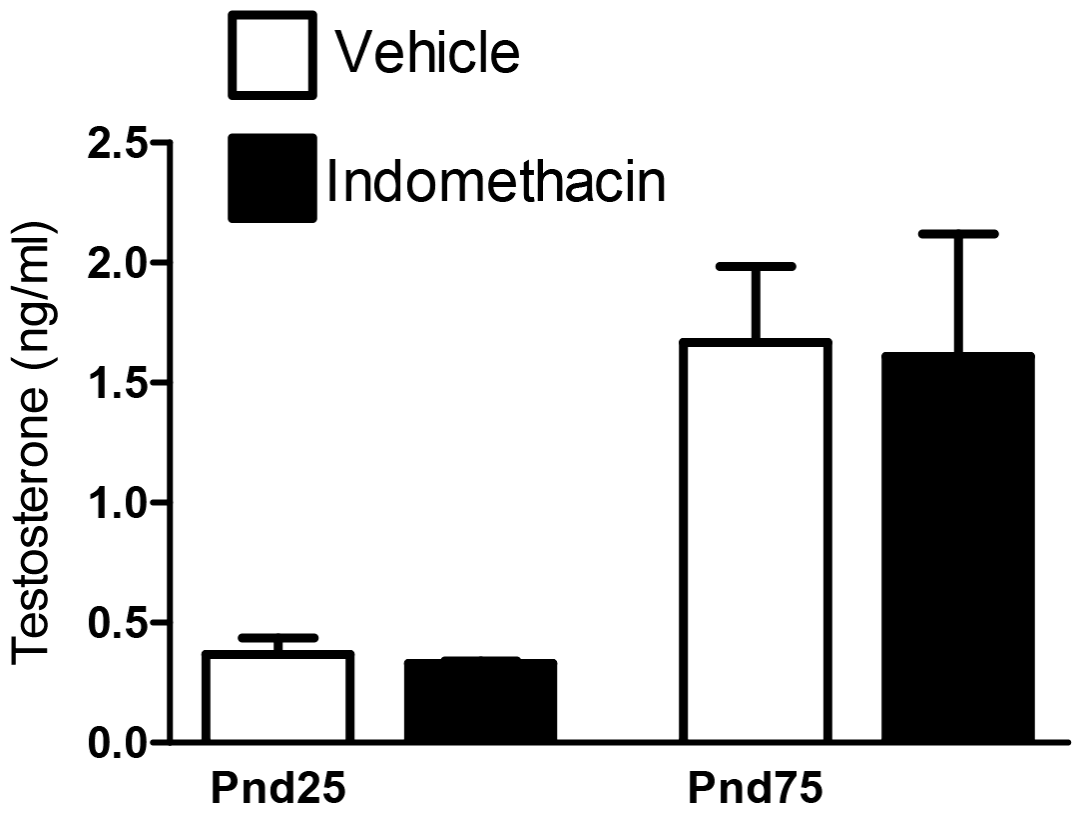
Effects of maternal exposure to vehicle or indomethacin (0.8 mg/kg/day; e15.5–e18.5) on serum testosterone levels at Pnd25 and Pnd75 (adulthood). Values are means ± SEM for N = 4–13 animals from a minimum of two litters.

By puberty (Pnd25), indomethacin exposed males were significantly heavier (∼24%) than vehicle exposed control animals, and this difference (∼23%) was still evident at Pnd75 (Fig. 3A). AGI at Pnd25 and Pnd75 was unaffected by fetal exposure to indomethacin (Fig. 3B) and in utero exposure to indomethacin did not significantly alter plasma levels of testosterone at either age (Fig. 4).

**Figure 5:**
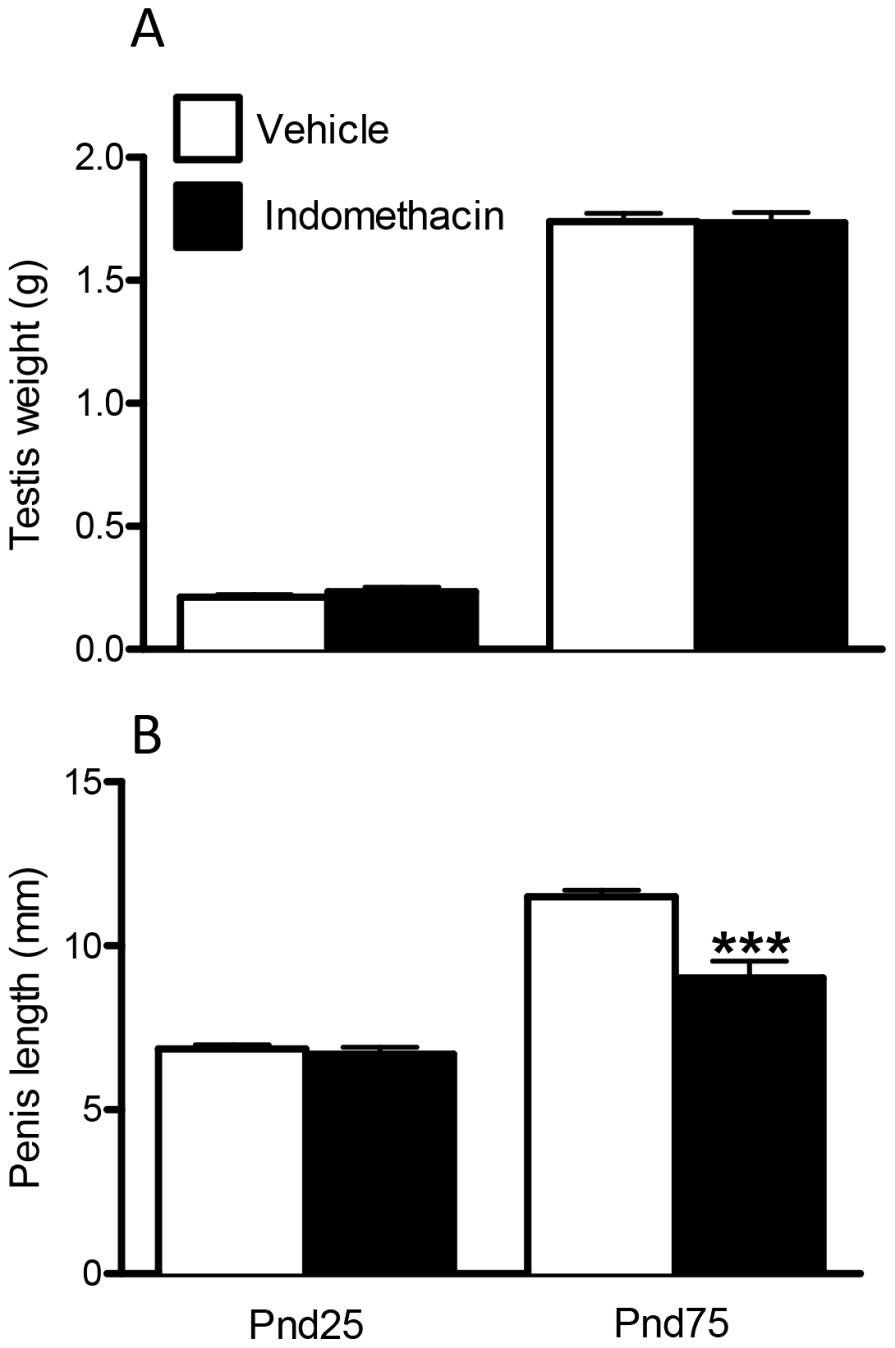
Effects of maternal exposure to vehicle or indomethacin (0.8 mg/kg/day; e15.5–e18.5) on testis weight (A), and penis length (B) at Pnd25 and Pnd75 (adulthood). Values are means ± SEM for N = 6–20 from a minimum of three litters. ***p<0.0001, in comparison with respective control.

Testis weight in indomethacin-exposed animals was comparable to controls at both ages (Fig. 5A) and there were no gross morphological changes in the testis (data not shown). No change was found in penile length at Pnd25 in indomethacin exposed animals but, unexpectedly, in adulthood, males exposed in utero to indomethacin, had a significantly shorter penile length (26% reduction) than controls (Fig. 5B).

## Discussion

Two lines of evidence prompted the present studies. The first was the clear demonstration in mice that exposure to PG inhibitors, such as indomethacin, during the MPW inhibited masculinization (AGD) [10],[11]. The second was evidence from human studies that exposure to acetaminophen (± other painkillers) during the presumptive MPW period increased incidence of cryptorchidism in male offspring [17]–[20], supported by rat experimental data that treatment with acetaminophen (during the MPW) reduced AGD [19]. A common pathway that might link these findings would be inhibition of PG production, although acetaminophen can also exert effects via other (incompletely understood) mechanisms [26]. We used the rat, in which we have established robust information on timing of the MPW and its susceptibility to disruption [4],[5],[25] to replicate earlier mouse studies [10],[11] and to identify whether interference with PG production/action during the MPW was likely to lead to disorders/impairment of masculinization. Our findings do not support such a role for PGs in the rat.

Despite replicating the treatment protocol of Gupta [10],[11], though with a slightly lower dose of indomethacin (0.8 mg/kg versus 1 mg/kg) because of concerns about confounding maternal toxic effects observed with the higher dose, we were unable to demonstrate any interference with masculinization, in terms of AGD/AGI or reproductive development. One obvious explanation would be that in rats indomethacin did not transfer across the placenta as readily as in mice. We cannot exclude this possibility completely, but our demonstration that indomethacin can decrease PGE_2_ in the fetal testis 4 h later suggests that placental transfer of indomethacin does occur. The decrease in PGE_2_ caused by indomethacin exposure during the MPW did not affect ITT levels during the MPW, nor plasma testosterone postnatally nor AGI, suggesting that PGE_2_ is not crucial for correct and full masculinization in rats. The stark difference in effects of indomethacin in the rat (present studies) and in the mouse [10],[11] is unexplained and is presumed to reflect a species difference. The absence of any effect of indomethacin on ITT in our studies, contrasts with studies in vitro that showed mild inhibition of testosterone production by e14.5 fetal rat testis cultures exposed to 10, but not to 1, micromolar indomethacin for 72 h [22]. This might indicate that higher levels of indomethacin exposure can suppress ITT, but to obtain higher exposure in vivo was not feasible in our rat studies because of maternal toxicity effects.

Some significant effects in males were seen after fetal indomethacin exposure, such as a decrease in testis weight at e21.5, although this corrected postnatally; we are currently investigating the cause of this reduction. One completely unexplained effect of in utero indomethacin exposure was the finding of a significant decrease in adult (final) penile length whereas penis length at Pnd25 was normal. Our previous studies have shown that penis length is programmed by androgen action in the MPW [5],[27],[28], although its growth after this time period is also androgen-dependent [27]. As ITT during the MPW and AGI at all ages was normal in indomethacin-exposed animals, and postnatal blood testosterone levels were normal at the two ages investigated, there is no obvious explanation for this finding. It is possible that fetal indomethacin exposure could have led to transient reduction in gonadotrophin secretion postpubertally, with the resulting lowering of testosterone leading to reduced penile growth, but a mechanism for such a change is not obvious. It should be noted also that, although penis length can vary in adult rats, the average length found in adult indomethacin-exposed animals lay below the range of values that we have seen in controls.

In conclusion, in utero indomethacin exposure did not result in overt inhibition of masculinisation nor the induction of recognizable masculinisation disorders in rats that occur with similar timed exposure to other compounds (e.g. flutamide, dibutyl phthalate) [5],[27] [29],[30]. Although some effects were found, such as a decrease in fetal testis weight and adult penile length, overall masculinisation was unaffected. In summary it can be concluded that exposure to indomethacin during the MPW, at a dose sufficient to cause a decrease in intra-testicular PGE_2_, is not involved in androgen driven masculinisation during the MPW. More detailed studies as to the role of PGs in penile development are required.

## Supporting Information

Figure S1 **Effect of maternal exposure to vehicle or indomethacin (0.8 mg/kg/day) on testicular PGE_2_ levels at e21.5 in rats.** Values are means ± SEM for N = 5. *p<0.05, in comparison with respective control. Treatment details are described in Materials & Methods.
(TIF)

## References

[1] Skakkebaek NE, Rajpert-De Meyts E, Main KM (2001) Testicular dysgenesis syndrome: an increasingly common developmental disorder with environmental aspects. Hum Reprod 16: 972–978. 11331648 10.1093/humrep/16.5.972

[2] Skakkebaek NE, Rajpert-De Meyts E, Jorgensen N, Main KM, Leffers H, et al (2007) Testicular cancer trends as ‘whistle blowers’ of testicular developmental problems in populations. Int J Androl 30: 198–204. 17705804 10.1111/j.1365-2605.2007.00776.x

[3] Sharpe RM, Skakkebæk NE (2008) Testicular dysgenesis syndrome: Mechanistic insights and potential new downstream effects. Fertil Steril 89: e33–38. 18308057 10.1016/j.fertnstert.2007.12.026

[4] Welsh M, Saunders PT, Fisken M, Scott HM, Hutchison GR, et al (2008) Identification in rats of a programming window for reproductive tract masculinization, disruption of which leads to hypospadias and cryptorchidism. J Clin Invest 118: 1479–1490. 18340380 10.1172/JCI34241 PMC2267017

[5] MacLeod DJ, Sharpe RM, Welsh M, Fisken M, Scott HM, Hutchison GR, et al (2010) Effect of disruption of androgen production or action in the masculinisation programming window on the development of male reproductive organs. Int J Androl 33: 279–287. 20002220 10.1111/j.1365-2605.2009.01005.x

[6] Swan SH, Main KM, Liu F, Stewart SL, Kruse RL, et al (2005) Decrease in anogenital distance among male infants with prenatal phthalate exposure. Environ Health Perspect 113: 1056–1061. 16079079 10.1289/ehp.8100 PMC1280349

[7] Thankamony A, Ong KK, Dunger DB, Acerini CL, Hughes IA (2009) Anogenital distance from birth to 2 years: a population study. Environ Health Perspect 117: 1786–1790. 20049133 10.1289/ehp.0900881 PMC2801188

[8] Hsieh MH, Breyer BN, Eisenberg ML, Baskin LS (2008) Associations among hypospadias, cryptorchidism, anogenital distance, and endocrine disruption. Curr Urol Rep 9: 137–142. 18419998 10.1007/s11934-008-0025-0

[9] Eisenberg ML, Hsieh MH, Walters RC, Krasnow R, Lipshultz LI (2011) The relationship between anogenital distance, fatherhood, and fertility in adult men. PLoS ONE 6: e18973. 21589916 10.1371/journal.pone.0018973 PMC3092750

[10] Gupta C, Goldman AS (1986) The arachidonic acid cascade is involved in the masculinizing action of testosterone on embryonic external genitalia in mice. Proc Natl Acad Sci USA 83: 4346–4349. 3086881 10.1073/pnas.83.12.4346 PMC323729

[11] Gupta C (1989) The role of prostaglandins in masculine differentiation: modulation of prostaglandin levels in the differentiating genital tract of the fetal mouse. Endocrinology 1: 124–129. 10.1210/endo-124-1-129 2521205

[12] Oates JA, FitzGerald GA, Branch RA, Jackson EK, Knapp HR, et al (1988) Clinical implications of prostaglandin and thromboxane A2 formation. N Engl J Med 319: 689–698. 3045550 10.1056/NEJM198809153191106

[13] Armstrong D (1981) Prostaglandins and follicular functions. J Reprod. Fert 62: 283–291. 10.1530/jrf.0.0620283 6262509

[14] Gaytán F, Tarradas E, Bellido C, Morales C, Sánchez-Criado JE (2002) Prostaglandin E_2_ inhibits abnormal follicle rupture and restores ovulation in indomethacin-treated rats. Biol Reprod 67: 1140–1147. 12297529 10.1095/biolreprod67.4.1140

[15] Amateau SK, McCarthy MM (2004) Induction of PGE2 by estradiol mediates developmental masculinization of sex behavior. Nat Neurosci 6: 643–650. 10.1038/nn1254 15156148

[16] Hannah ME, Ohlsson A, Farine D, Hewson SA, Hodnett ED, et al (1996) Induction of labor compared with expectant management for prelabor rupture of the membranes at term. N Eng J Med 334: 1005–1010. 10.1056/NEJM199604183341601 8598837

[17] Berkowitz GS, Lapinski RH (1996) Risk factors for cryptorchidism: a nested case-control study. Paediatr Perinat Epidemiol 10: 39–51. 8746430 10.1111/j.1365-3016.1996.tb00024.x

[18] Jensen MS, Rebordosa C, Thulstrup AM, Toft G, Sørensen HT, et al (2010) Maternal use of acetaminophen, ibuprofen, and acetylsalicylic acid during pregnancy and risk of cryptorchidism. Epidemiology 21: 779–85. 20805751 10.1097/EDE.0b013e3181f20bed

[19] Kristensen D, Hass U, Lesne L, Lottrup G, Jacobsen PR, et al (2010) Intrauterine exposure to mild analgesics is a risk factor for development of male reproductive disorders in human and rat Human Reproduction,. Vol.0: 1–10. 10.1093/humrep/deq323 21059752

[20] Snijder CA, Kortenkamp A, Steegers EA, Jaddoe VW, Hofman A, et al (2012) Intrauterine exposure to mild analgesics during pregnancy and the occurrence of cryptorchidism and hypospadia in the offspring: the Generation. R Study Hum Reprod 27: 1191–1201. 22301570 10.1093/humrep/der474

[21] Amann RP, Veeramachaneni DNR (2006) Cryptorchidism and associated problems in animals. Anim Reprod 3: 108–120.

[22] Kristensen DM, Lesné L, Le Fol V, Desdoits-Lethimonier C, Dejucq-Rainsford N, et al (2012) Paracetamol (acetaminophen), aspirin (acetylsalicylic acid) and indomethacin are anti-androgenic in the rat foetal testis. Int J Androl 3: 377–84. 10.1111/j.1365-2605.2012.01282.x 22612476

[23] Dean A, Smith LB, Macpherson S, Sharpe RM (2012) The effect of dihydrotestosterone exposure during or prior to the masculinization programming window on reproductive development in male and female rats. Int J Androl 35: 330–339. 22248293 10.1111/j.1365-2605.2011.01236.x

[24] Van den Driesche S. Walker M, Smith LB, Anderson RA, Drake AJ, et al (2012) Proposed role for COUP-TFII in regulating fetal Leydig cell steroidogenesis, perturbation of which leads to masculinization disorders in rodents. PloS One 7: e37064. 22615892 10.1371/journal.pone.0037064 PMC3355148

[25] Drake AJ, van den Driesche S, Scott HM, Hutchison GR, Seckl JR, et al (2009) Glucocorticoids amplify dibutyl phthalate-induced disruption of testosterone production and male reproductive development. Endocrinology 150: 5055–5064. 19819957 10.1210/en.2009-0700

[26] Graham GG, Scott KF (2005) Mechanism of action of paracetamol. Am J Ther 12: 46–55. 15662292 10.1097/00045391-200501000-00008

[27] Van den Driesche S, Scott HM, Macleod DJ, Fisken M, Walker M, et al (2011) Relative importance of prenatal and postnatal androgen action in determining growth of the penis and anogenital distance in the rat before, during and after puberty. Int J Androl 34: e578–e586. 21631528 10.1111/j.1365-2605.2011.01175.x

[28] Welsh M, MacLeod DJ, Walker M, Smith LB, Sharpe RM (2009) Critical androgen-sensitive periods of rat penis and clitoris development. Int J Androl 33: 144–152. 10.1111/j.1365-2605.2009.00978.x PMC2816361 19656234

[29] Mylchreest E, Wallace DG, Cattley RC, Foster PMD (2000) Dose-dependent alterations in androgen-regulated male reproductive development in rats exposed to Di(n-butyl) phthalate during late gestation. Tox Sci 55: 143–151. 10.1093/toxsci/55.1.143 10788569

[30] Parks LG, Ostby JS, Lambright CR, Abbott BD, Klinefelter GR, et al (2000) The plasticizer diethylhexyl phthalate induces malformations by decreasing fetal testosterone synthesis during sexual differentiation in the male rat. Tox Sci 58: 339–349. 10.1093/toxsci/58.2.339 11099646

