# Supplementary figures and images for: Prostaglandins, Masculinization and Its Disorders: Effects of Fetal Exposure of the Rat to the Cyclooxygenase Inhibitor- Indomethacin
Source: PLoS One. 2013 May 3;8(5):e62556. doi: 10.1371/journal.pone.0062556 (PMC3643956; doi:10.1371/journal.pone.0062556)

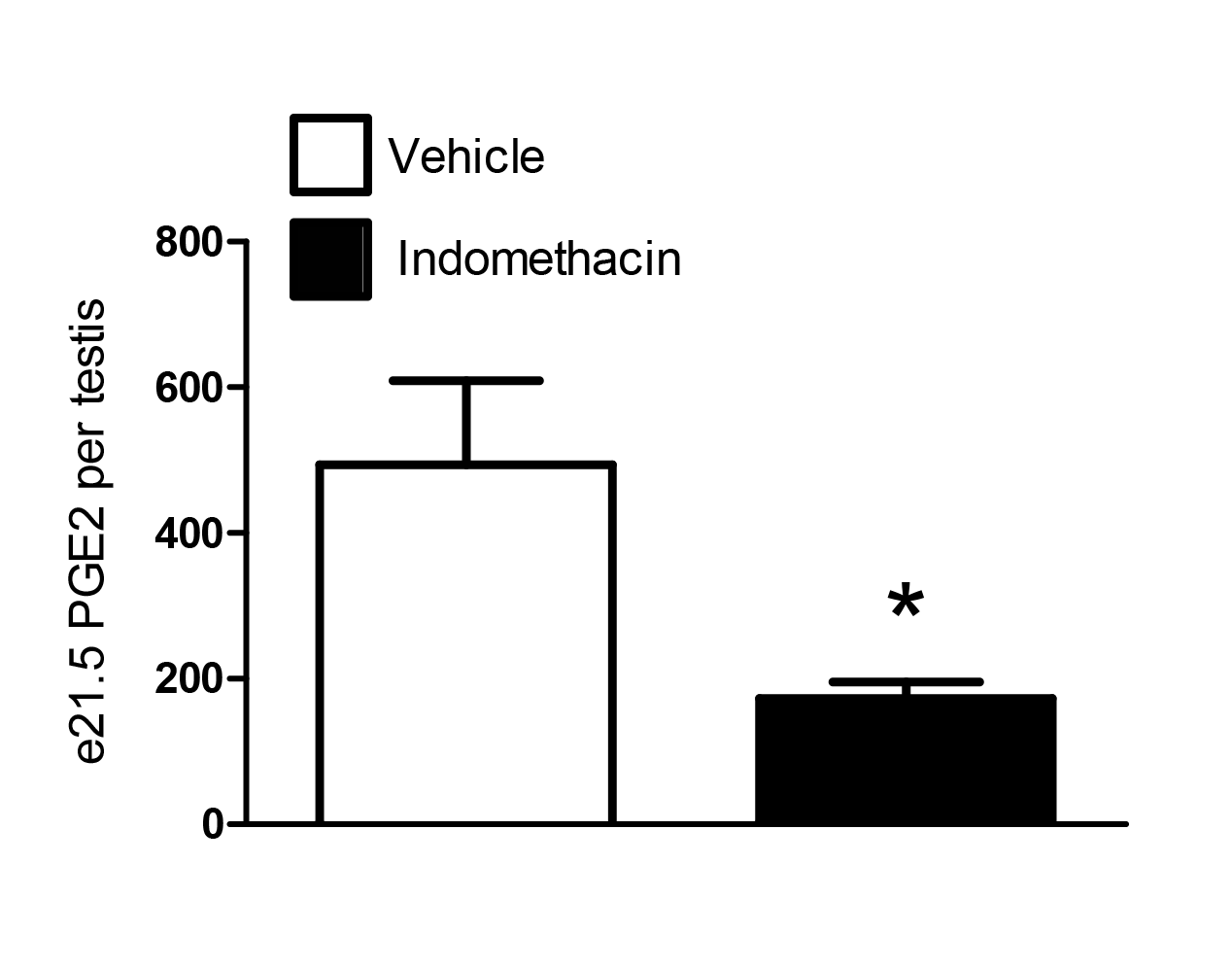

Supplement: Figure S1 — Effect of maternal exposure to vehicle or indomethacin (0.8 mg/kg/day) on testicular PGE2 levels at e21.5 in rats. Values are means ± SEM for N = 5. *p<0.05, in comparison with respective control. Treatment details are described in Materials & Methods. (TIF) [file pone.0062556.s001.tif]
